# A Novel Noninvasive Diagnostic Model of HBV-Related Inflammation in Chronic Hepatitis B Virus Infection Patients With Concurrent Nonalcoholic Fatty Liver Disease

**DOI:** 10.3389/fmed.2022.862879

**Published:** 2022-03-23

**Authors:** Xuemei Tao, Lin Chen, Youfei Zhao, Yonggang Liu, Ruifang Shi, Bei Jiang, Yuqiang Mi, Liang Xu

**Affiliations:** ^1^Clinical School of the Second People's Hospital, Tianjin Medical University, Tianjin, China; ^2^Department of Hepatology, Tianjin Second People's Hospital, Tianjin, China; ^3^Tianjin Research Institute of Liver Diseases, Tianjin, China

**Keywords:** hepatitis B, nonalcoholic fatty liver, inflammation, noninvasive diagnosis, model

## Abstract

**Background and Aims:**

Patients with chronic hepatitis B virus infection (CBI) with concurrent nonalcoholic fatty liver disease (NAFLD) is becoming increasingly common in clinical practice, and it is quite important to identify the etiology when hepatitis occurs. A noninvasive diagnostic model was constructed to identify patients who need antihepatitis B virus (HBV) therapies [histologic activity index (HAI) ≥ 4] in patients with CBI with concurrent NAFLD by analyzing clinical routine parameters.

**Approach and Results:**

In total, 303 out of 502 patients with CBI with concurrent NAFLD proven by liver biopsy from January 2017 to December 2020 in the Tianjin Second People's Hospital were enrolled and they were divided into the HBV-related inflammation (HBV-I) group (HAI ≥ 4,176 cases) and the non-HBV-I group (HAI < 4,127 cases) according to hepatic pathology. The univariate analysis and multivariate logistic regression analysis were performed on the two groups of patients, and then the HBV-I model of patients with CBI with concurrent NAFLD was constructed. The areas under receiver operating characteristic curves (AUROCs) were used to evaluate the parameters of the regression formula. Another 115 patients with CBI with concurrent NAFLD proven by liver biopsy from January 2021 to January 2022 were enrolled as the validation group. There were some statistical differences in demographic data, biochemical indicators, immune function, thyroid function, virology indicator, and blood routine indicators between the two groups (*P* < 0.05) and liver stiffness measurement (LSM) in the HBV-I group was significantly higher than those in the non-HBV-I group (*P* < 0.05). While controlled attenuation parameters (CAP) in the HBV-I group were lower than those in the non-HBV-I group (*P* < 0.05); (2) We developed a novel model by logistic regression analysis: HBV-I = −0.020 × CAP + 0.424 × LSM + 0.376 × lg (HBV DNA) + 0.049 × aspartate aminotransferase (AST) and the accuracy rate was 82.5%. The area under the receiver operating characteristic (AUROC) is 0.907, the cutoff value is 0.671, the sensitivity is 89.30%, the specificity is 77.80%, the positive predictive value is 90.34%, and the negative predictive value is 81.89%; (3) The AUROC of HBV-I in the validation group was 0.871 and the overall accuracy rate is 86.96%.

**Conclusion:**

Our novel model HBV-I [combining CAP, LSM, lg (HBV DNA), and AST] shows promising utility for predicting HBV-I in patients with CBI with concurrent NAFLD with high sensitivity, accuracy, and repeatability, which may contribute to clinical application.

## Introduction

With the rapid development of anti-hepatitis B virus (HBV) drugs, the popularization of preventing mother-to-child transmission (PMTCT) of HBV, and the standardized management of blood donation and organ transplantation, the rate of new HBV infections has dropped significantly. However, HBV infection is still prevalent worldwide without any cure methods currently. According to the WHO (1), 2 billion people have been infected with HBV worldwide, of which 250 million are chronic carriers of HBV, and approximately 800,000 people die from cirrhosis or hepatocellular carcinoma (HCC) caused by chronic hepatitis B virus infection (CBI) each year. 8-20% of patients with chronic hepatitis B (CHB) can progress to cirrhosis, and within 5 years, 2–8% of those with cirrhosis advance to HCC (2). Recently, it is estimated that the prevalence of hepatitis B surface antigen (HBsAg) in China is 5–6%, of which about 20 million to 30 million cases of patients with CHB (3).

Meanwhile, the incidence of nonalcoholic fatty liver disease (NAFLD) has increased with years since the improvement of living standards and lifestyle changes, which is recognized as the leading causes of chronic liver disease worldwide (4). NAFLD is an acquired metabolic liver injury, which is closely associated with obesity, insulin resistance, and hyperlipidemia, and the global prevalence of NAFLD is ~25% (5). It is estimated that at present the overall prevalence of NAFLD in Asia is 29.6% (6). Additionally, the prevalence of NAFLD in China, it is considered (7), is approximately 29.88%. NAFLD (8) encompasses a spectrum of diseases, including nonalcoholic fatty liver (NAFL), nonalcoholic steatohepatitis (NASH), and related cirrhosis.

Many studies (9, 10) have shown that concurrent with NAFLD can accelerate the course progression of patients with CBI in hepatic fibrosis, cirrhosis, and even HCC. When abnormal liver function occurs in these patients, such as elevated alanine aminotransferase (ALT) (11), it is important to identify the cause of the inflammation for making the therapeutic scheme. At present, the gold standard of clinical identification is still liver biopsy, however, there are numerous shortcomings for biopsy, such as invasiveness, high price, and poor reproducibility. Furthermore, we have searched relevant literature in recent years and there is still no ideal noninvasive diagnosis method. To fill this knowledge gap, we intend to develop and validate a noninvasive diagnostic model based on common clinical data in this article, which has been certain instructive to identify HBV-related inflammation (HBV-I) in patients of CBI concurrent with NAFLD.

## Materials and Methods

### Patients and Inclusion/Exclusion Criteria

All the subjects in the modeling group and the validation group had provided informed consent for their medical information to be used for this research and were approved by the Medical Ethics Committee of the Tianjin Second People's Hospital.

**Figure 1:**
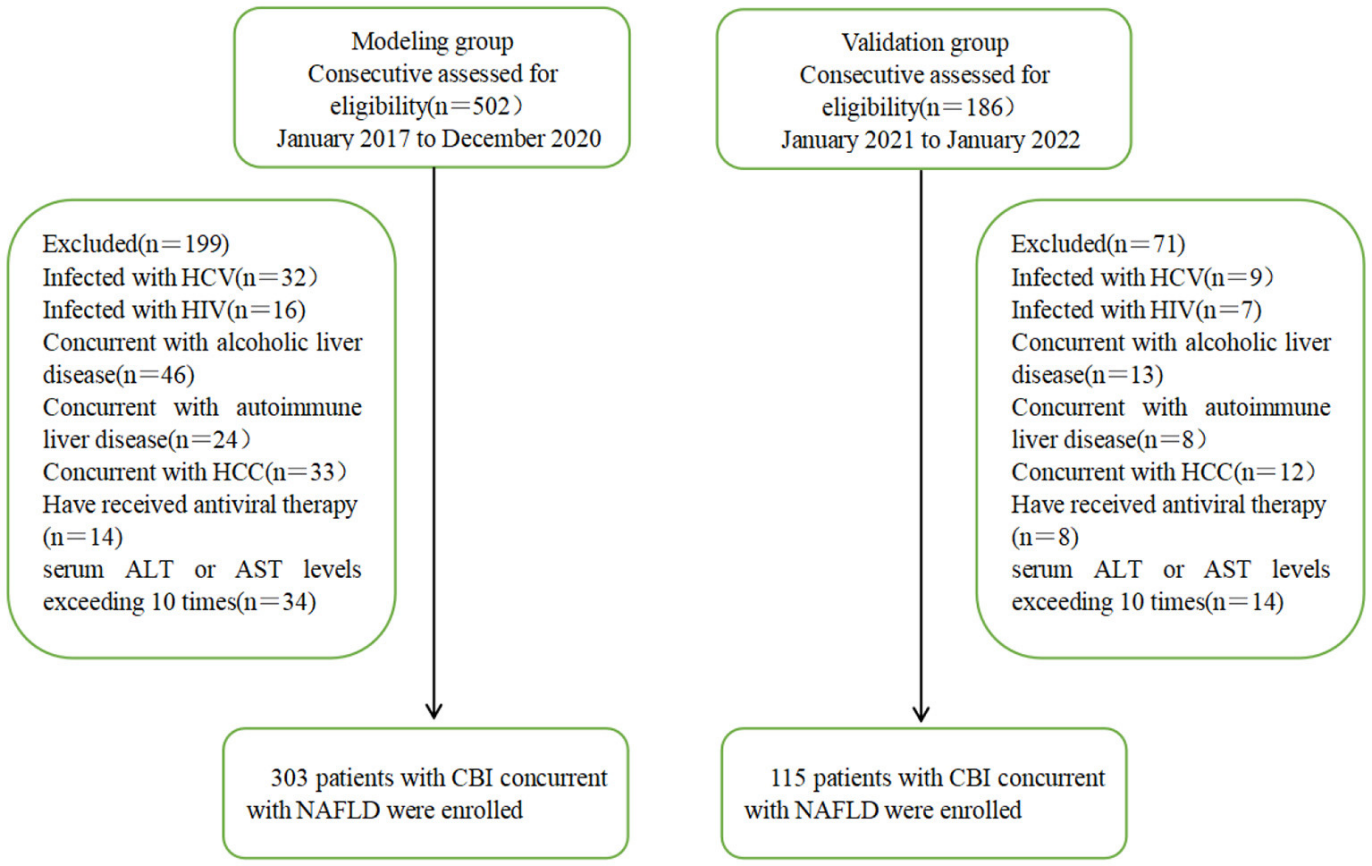
Flowchart of patient recruitment in the modeling and validation groups. HCV, hepatitis C virus; HIV, Human Immunodeficiency Virus; HCC, Hepatocellular Carcinoma; ALT, alanine aminotransferase; AST, aspartate aminotransferase; CBI, chronic HBV infection; NAFLD, nonalcoholic fatty liver disease.

A total of 303 patients with CBI with concurrent NAFLD who were hospitalized and liver biopsy-proven in the Tianjin Second People's Hospital from January 2017 to December 2020 were enrolled in this study. They were divided into HBV-I group (12) (histologic activity index, HAI ≥ 4) in patients with CBI with concurrent NAFLD, 176 cases) and non-HBV-I group (HAI < 4 in patients with CBI with concurrent NAFLD, 127 cases) according to liver histopathology. Another 115 patients with CBI with concurrent NAFLD proven by liver biopsy in Tianjin Second People's Hospital from January 2021 to January 2022 were enrolled as the validation group. Inclusion criteria: (1) in accordance with the Chinese Medical Association's “Guidelines for the Prevention and Treatment of Chronic Hepatitis B (Updated in 2019)” (13) to diagnose CBI; (2) The diagnosis of NAFLD and NASH complies with the 2018 NAFLD Diagnosis and Treatment Guidelines (14). Exclusion criteria: (1) concurrent with other viral hepatitis, such as types A, C, D, and E; (2) concurrent with other liver diseases, such as alcoholic liver disease, autoimmune liver disease, drug-induced liver injury, and hepatolenticular nucleus; (3) concurrent with liver cancer or malignant tumors from other systems; (4) Have received antiviral therapy or long-term treatment with immunosuppressants and glucocorticoids; (5) concurrent with thyroid disease or blood system disease; (6) concurrent with human immunodeficiency virus or other autoimmune diseases; (7) ALT or aspartate aminotransferase (AST) levels exceeding 10 times the upper limit of normal (ULN). Of 502 participants assessed for eligibility in the modeling group, 303 CBI patients with concurrent NAFLD in inflammation fulfilled the inclusion criteria, whereas 115 of 186 subjects were eligible in the validation group. A flow diagram outlining the patient selection is shown in Figure 1.

### Clinical and Laboratory Data

Demographic data (gender, age, height, and weight) of the two groups were collected, and their body mass index (BMI) was calculated. A blood sample was collected from each subject after 12-h fasting, and automatic biochemical analyzer-7180 (HITACH1, Japan) was used to detect the patient's biochemical indicators, including ALT, AST, γ-alanine transferase (γ-GT), Alkaline phosphatase (ALP), total protein (TP), albumin (ALB), total bilirubin (TBIL), urea nitrogen (BUN), blood creatinine (Ccr), uric acid (UA), triglyceride (TG), total cholesterol (CHO), high-density lipoprotein (HDL), low-density lipoprotein (LDL), and fasting blood glucose (FBG); four iron items: transferrin (TFR), serum iron (Fe), ferritin (FER), total iron-binding capacity (TIBC), and unsaturated iron-binding capacity (UIBC); immune function: complement 3 (C3), complement 4 (C4), immunoglobulin G (IgG), immunoglobulin A (IgA), and immunoglobulin (IgM). An automatic electrochemiluminescence analyzer (Roche COBAS e 411, Sandhofer Strasse 116, 68305 Mannheim, Germany) was used to detect patient's thyroid function: triiodothyronine (tt3), tetraiodothyronine (tt4), free triiodothyronine (FT3), free tetraiodothyronine (FT4), thyroid-stimulating hormone (TSH), thyroglobulin Binding antibody (TGAB); five hepatitis B items: HBsAg, hepatitis B surface antibody (HBsAb), hepatitis B e antigen (HBeAg), hepatitis B e antibody (HBeAb), hepatitis B core antibody (HBcAb). HBV-DNA quantification was detected by PCR. Automatic blood analyzer XN-2000 (Sysmex, Japan) was used to detect the patient's blood routine indicators: platelets (PLT), white blood cells (WBC), neutrophils (NEUT), lymphocytes (LYMPH), red blood cell (RBC), hemoglobin (HGB), hematocrit (HCT), mean red blood cell volume (MCV), and mean platelet volume (MPV).

### FibroScan Inspection

Liver stiffness measurement (LSM, in kPa) and controlled attenuation parameter (CAP, in dB/m) were measured in 1 week before liver biopsy using FibroScan-502 (Echosens, Paris, France) operated by professionals in our hospital and took the median of 10 measurements and the ratio of the interquartile range (IQR) to the median (IQR/med) of the measured value is required to be <30%, and the success rate (successful detection times/total detection times) ≥ 60% is a valid measurement. The detection range is 7-9 intercostal from the right anterior axillary line to the media axillary line.

### Liver Pathology and Definition of Different Inflammation Groups

Liver biopsy was performed under the guidance of B-ultrasound using an 18G bard biopsy needle. The liver tissue specimen requirements are length > 1.5 cm, including ≥4 complete manifold areas. After 10% formaldehyde fixation, paraffin embedding, and sectioning, pathological examinations were performed.

The Knodell HAI (12) was used to describe the intensity of hepatocellular necroinflammatory activity [the HAI scores consist of the combined score of the four categories in a liver biopsy specimen: (a) periportal ± bridging hepatocellular necrosis; (b) intralobular degeneration and focal hepatocellular necrosis; (c) portal inflammation, and (d) fibrosis; HAI ≥ 4 named the HBV-I group, whereas HAI < 4 named the non-HBV-I group], and the diagnostic criteria of the degree of fibrosis (F, graded from 0 to 4, F0 = no fibrosis, F1 = portal fibrosis without septa, F2 = portal fibrosis with rare septa, F3 = numerous septa without cirrhosis, and F4 = cirrhosis) are based on the Metavir scoring system (15). Hepatic steatosis was graded according to the percentage of hepatocytes affected: none (<5%), mild steatosis (5–32%), moderate steatosis (33–65%), and severe steatosis (≥66%) (14). According to the NAFLD Activity Score (NAS) recommended by the Pathology Committee of the NASH Clinical Research Network (16), the score is defined as the unweighted sum of the scores for steatosis (0–3), lobular inflammation (0–3), and ballooning (0–2); thus ranging from 0 to 8. NAS of >4 correlated with a diagnosis of NASH and biopsies with scores of ≤4 were diagnosed as NAFL. According to the working definition of CBI concurrent with NAFLD liver inflammation, patients with HAI ≥4 were divided into the HBV-I group (176 cases) and patients with HAI < 4 were divided into the non-HBV-I group (127 cases), without regard to the NAS score.

**Figure 2:**
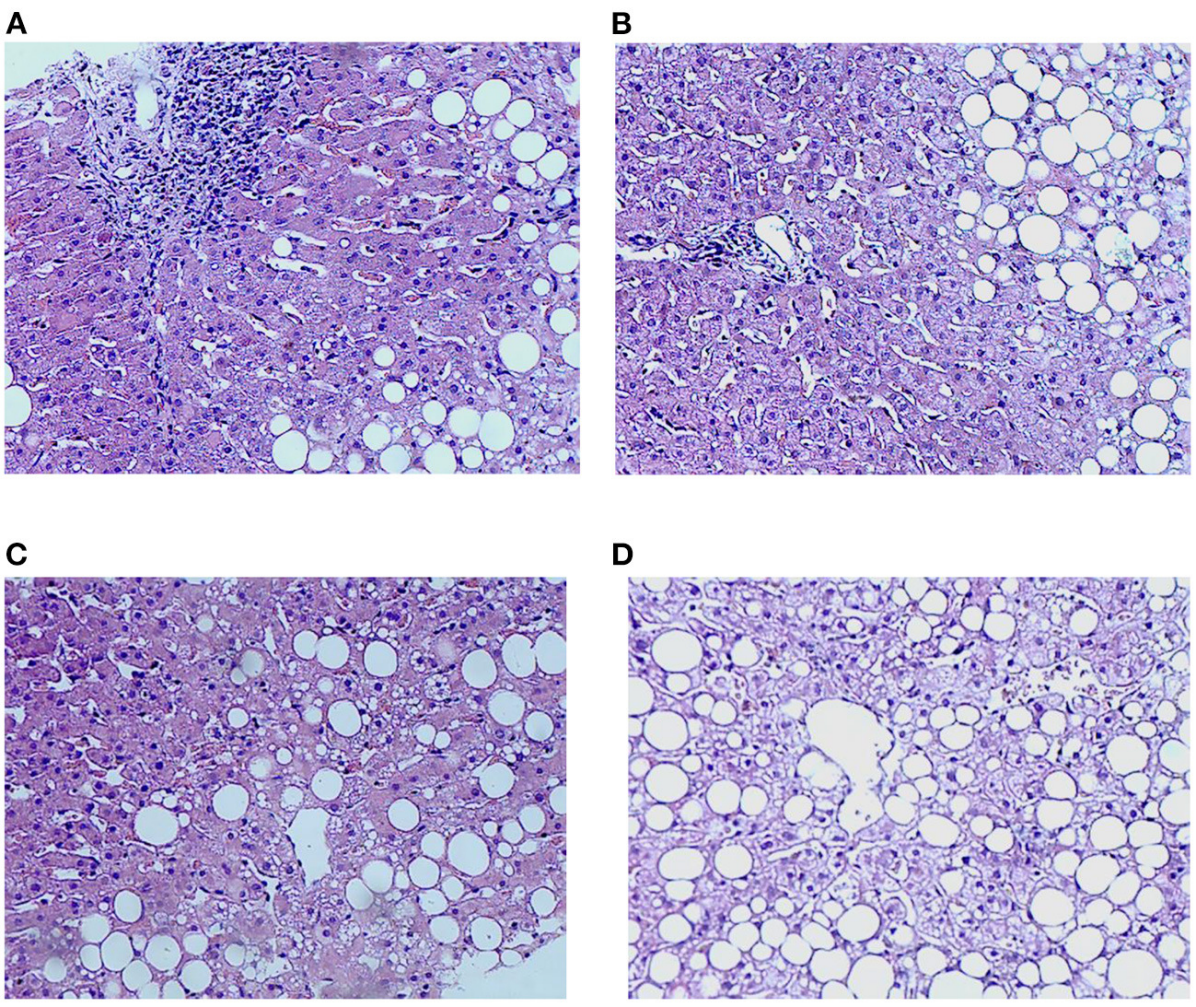
Characteristics of inflammation and lipidosis in the liver between the HBV-RI group and the non-HBV-RI group. HBV-RI group, HBV-related inflammation group; non-HBV-RI group, non-HBV-related inflammation group. **(A)** Pathological characteristics of inflammation in the HBV-related inflammation group (HE 200 ×); **(B)** pathological characteristics of inflammation in the non-HBV-related inflammation group (HE 200 ×): no obvious inflammation and no boundary plate damage in the portal area (HE 200 ×); **(C)** lipidosis characteristics of the HBV-related inflammation group (HE 200 ×); **(D)** lipids in the non-HBV-related inflammation group (HE 200 ×).

Liver pathology is independently diagnosed by two pathologists above the primary level in the pathology department blinded to the clinical data and noninvasive tests. When inconsistencies occurred, the examination should be repeated till to reach a consensus (outlined in Figure 2).

### Establishment of a Noninvasive Diagnostic Model

Those clinical variables with *P*-value < 0.2 between the two groups were entered into the multivariate analysis and then binary logistic regression analysis was performed; additionally, age and gender were adjusted to establish a noninvasive diagnostic model.

### Statistical Analysis

SPSS statistical software (SPSS Incorporation, Chicago, Illinois, USA, version 25.0 for Windows) was used to analyze the data. Measurement data conforming to normal distribution are expressed as ($\bar{x}$ ± *s*), and HBV DNA was calculated by denary logarithm. Intergroup comparisons were calculated using the *t*-test. Data with non-normal distribution were expressed as the median (IQR 25–75%). and comparisons were performed using the Mann–Whitney *U*-test. Comparison of data variables between the groups was carried out by the chi-square test. Liver biopsy was used as the gold standard, and clinical indicators with differences between two groups were analyzed by univariate and multivariate logistic regression analysis, and the areas under the receiver operating characteristics (AUROCs) curves were used to evaluate the parameters of the regression formula. The cutoff value, sensitivity, and specificity were calculated by maximizing the Youden index (sensitivity + specificity – 1) to evaluate the diagnostic performance of CAP and LSM. GraphPad Prism 8 software was used for constructing figures. *P* < 0.05 was considered to indicate statistical significance for all the analyses.

## Results

### Baseline Demographics and Laboratory Data

**Figure 3:**
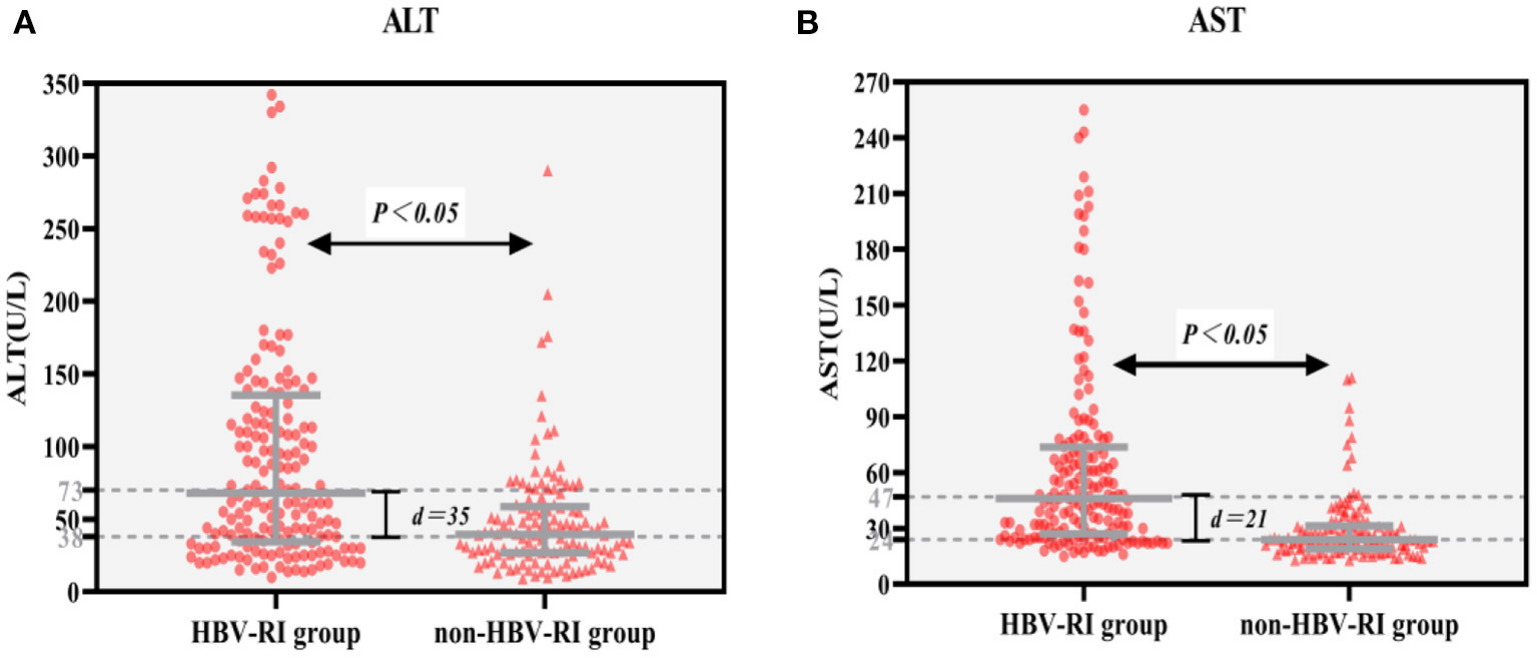
**(A,B)** Serum ALT and AST between the HBV-RI group and the non-HBV-RI group. HBV-RI group, HBV-related inflammation group; non-HBV-RI group, non-HBV-related inflammation group; ALT, alanine aminotransferase; AST, aspartate aminotransferase; The median value of ALT in the HBV-related inflammation group was 73 U/L, whereas in the non-HBV-related inflammation group was 38 U/L, and the difference was significant (*P* < 0.05); The median value of AST in the HBV-related inflammation group was 47 U/L, whereas in the non-HBV-related inflammation group was 24 U/L, and the difference was significant (*P* < 0.05).

Of 303 patients who fulfilled the inclusion criteria in the study divided according to HAI into HBV-I group and non-HBV-I group. No significance in clinical characteristics of age, BMI, TP, TBIL, BUN, CRE, CHO, HDL, LDL, and GLU were found between the two groups. However, the female patients in the HBV-I group were more than those in the non-HBV-I group, and the difference was statistically significant (*P* < 0.05). In addition, ALT, AST, γ-GT, and ALP in the HBV-I group were significantly higher than those in the non-HBV-I group (*P* < 0.05), while ALB, UA, and TG were significantly lower than those in the non-HBV-I group (*P* < 0.05), shown in Table 1 and Figure 3.

**Table 1:** Demographic and biochemical data between the HBV-RI group and the non-HBV-RI group.

**Variables**	**HBV-RI group** **(*n* = 176)**	**Non-HBV-RI group (*n* = 127)**	***t/Z/χ^2^***	***P***
Age (years)	38.54 ± 10.85	37.98 ± 9.96	0.458	0.647
Gender (M /F)	125/51	105/22	5.479	0.019
BMI (kg/m^2^)	27.90 ± 5.15	27.49 ± 3.61	0.627	0.531
ALT (U/L)	98.00 ± 85.54	48.73 ± 39.49	6.709	0.000
AST (U/L)	63.55 ± 54.58	29.07 ± 17.63	7.829	0.000
γ-GT(U/L)	51.50(27.00,83.5)	38.00(27.00,83.00)	−4.471	0.000
ALP (U/L)	79.13 ± 31.01	69.91 ± 24.90	2.693	0.008
TP (g/L)	73.16 ± 4.97	74.30 ± 5.04	−1.908	0.058
ALB (g/L)	45.16 ± 4.14	47.42 ± 3.58	−4.867	0.000
TBIL (μmol/L)	16.87 ± 7.85	15.20 ± 6.42	1.948	0.052
BUN (mmol/L)	4.56 ± 1.28	4.76 ± 1.09	−1.417	0.157
Ccr (μmol/L)	65.66 ± 15.18	68.20 ± 12.78	−1.493	0.137
UA (mmol/L)	340.63 ± 87.27	376.59 ± 91.09	−3.369	0.001
TG (mmol/L)	1.38 ± 0.68	1.79 ± 1.10	−3.527	0.001
CHO (mmol/L)	4.71 ± 0.95	4.73 ± 0.80	−0.180	0.857
HDL (mmol/L)	1.16 ± 0.29	1.11 ± 0.30	1.284	0.200
LDL (mmol/L)	2.78 ± 0.76	2.71 ± 0.65	0.739	0.461
FBG (mmol/L)	5.93 ± 1.36	6.09 ± 1.54	−0.908	0.364

*HBV-RI group, HBV-related inflammation group; non-HBV-RI group, Non-HBV-related inflammation group; numerical variables were shown as ($\bar{x}$ ± s) or M (P25, P75); categorical variables were expressed as n (%); M, male; F, female; BMI, body mass index; ALT, alanine aminotransferase; AST, aspartate aminotransferase; γ-GT, γ-alanine transferase; ALP, Alkaline phosphatase; TP, total protein; ALB, albumin; TBIL, total bilirubin; BUN, urea nitrogen; Ccr, blood creatinine; UA, uric acid; TG, triglyceride; CHO, total cholesterol; HDL, high-density lipoprotein; LDL, low-density lipoprotein; FBG, fasting blood glucose*.

**Figure 4:**
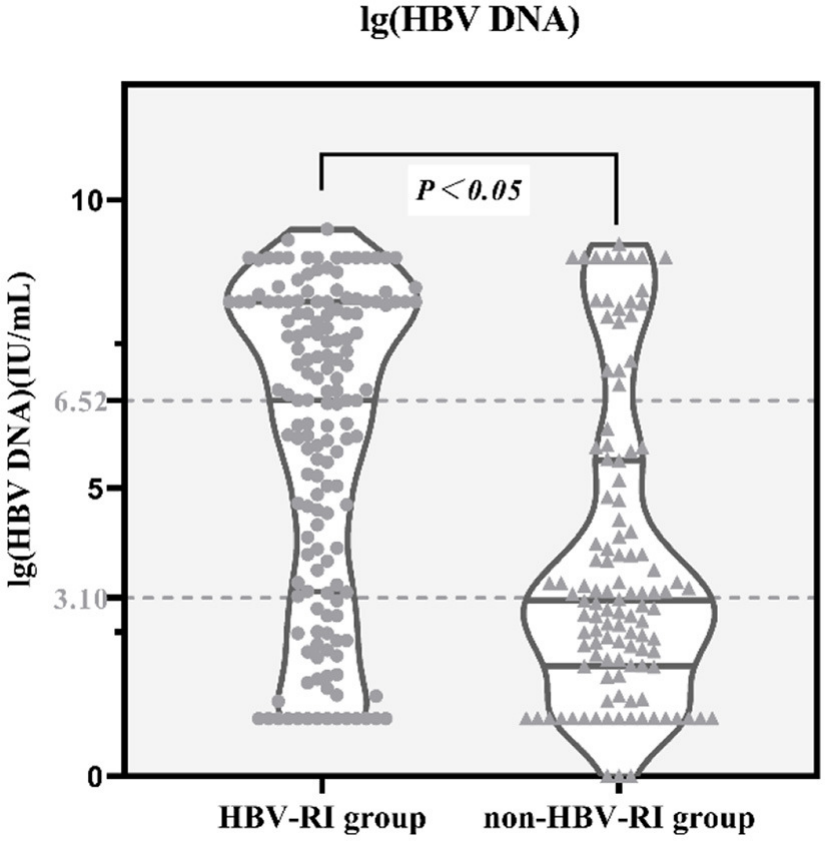
lg(HBV DNA) between the HBV-RI group and the non-HBV-RI group. HBV-RI group, HBV-related inflammation group; non-HBV-RI group, Non-HBV-related inflammation group; the median value of lg (HBV DNA) (HBV DNA was calculated by denary logarithm) in the HBV-related inflammation group was 6.52 IU/L, whereas in the non-HBV-related inflammation group was 3.10 IU/L, and the difference had statistically significant (*P* < 0.05).

Comparing the parameters of virological, thyroid function, immune function, and the blood routine test between the two groups, it was found that the positive rates of lg (HBV DNA), HBeAg, TT3, TT4, IgG, IgA, MCV, MPV in the HBV-I group were significantly higher than those in the non-HBV-I group (*P* < 0.05), while C3 and RBC were as significantly lower than the non-HBV-I group (*P* < 0.05), and there was no significant difference in the comparison of other parameters. See Table 2 and Figure 4 for details.

**Table 2:** Parameters of virological, thyroid function, immune function, and blood routine test between the HBV-RI group and the non-HBV-RI group.

**Variables**	**HBV-RI group** **(*n* = 176)**	**Non-HBV-RI group (*n* = 127)**	***t/Z/χ^2^***	***P***
HBsAg (IU/ml)	3890.32 (1741.2,15754.51)	2788.00 (731.83,35450.00)	−0.543	0.587
HBeAg ( + )	100/176	55/127	5.389	0.020
HBeAb ( + )	71/176	51/127	0.001	0.974
lg (HBV DNA)(IU/ml)	5.79 ± 2.71	3.75 ± 2.60	6.226	0.000
TT_3_ (nmol/L)	2.04 ± 0.50	1.88 ± 0.37	2.412	0.017
TT_4_ (nmol/L)	111.98 ± 26.65	104.41 ± 21.62	2.029	0.044
FT_3_ (pmol/L)	5.32 ± 2.46	5.07 ± 0.68	0.998	0.319
FT_4_ (pmol/L)	16.00 ± 2.23	16.29 ± 2.01	1.014	0.312
TSH (uIU/ml)	2.20 ± 1.52	2.13 ± 1.54	0.370	0.712
TGAB (IU/ml)	29.11 (24.46,34.37)	28.25 (20.06,52.01)	−0.935	0.350
TPOAB (IU/ml)	11.57 (8.86,15.26)	12.28 (9.92,15.51)	−0.486	0.627
C3 (g/L)	1.16 ± 0.28	1.24 ± 0.22	−2.583	0.010
C4 (g/L)	0.21 ± 0.09	0.23 ± 0.08	−1.637	0.103
IgG (g/L)	14.27 ± 4.15	13.21 ± 3.39	2.140	0.033
IgA (g/L)	2.87 ± 1.37	2.54 ± 1.02	2.179	0.030
IgM (g/L)	1.13 ± 0.56	1.00 ± 0.47	1.872	0.062
WBC (10^9^/L)	5.71 ± 1.68	5.92 ± 1.60	−1.057	0.291
NEUT (10^9^/L)	3.27(1.82,3.80)	3.01(2.36,3.46)	−0.486	0.627
LYMPH (10^9^/L)	1.85 ± 0.64	1.85 ± 0.58	−0.085	0.933
RBC (10^12^/L)	4.90 ± 0.56	5.07 ± 0.58	−2.536	0.012
HGB (g/L)	150.07 ± 16.88	153.45 ± 14.84	−1.806	0.072
HCT (%)	43.93 ± 6.15	44.90 ± 3.88	−1.569	0.118
MCV (fl)	89.82 ± 4.87	88.31 ± 4.97	2.638	0.009
PLT (10^9^/L)	205.40 ± 77.41	215.56 ± 52.16	−1.362	0.174
MPV (fl)	10.62 ± 1.02	10.28 ± 0.82	3.106	0.002

*HBV-RI group, HBV-related inflammation group; non-HBV-RI group, Non-HBV-related inflammation group; numerical variables were shown as ($\bar{x}$ ± s) or M (P25, P75); categorical variables were expressed as n (%); TFR, four iron items: transferrin; Fe, serum iron; FER, ferritin; TIBC, total-iron binding capacity; UIBC, unsaturated iron binding capacity; C3, complement 3; C4, complement 4; IgG, immunoglobulin G; IgA, immunoglobulin A; IgM, immunoglobulin; TT3, Triiodothyronine; TT4, Tetraiodothyronine; FT3, Free Triiodothyronine; FT4, Free Tetraiodothyronine; TSH, Thyroid Stimulating Hormone; TGAB, Thyroglobulin Binding Antibody; HBsAg, hepatitis B surface antigen; HBsAb, hepatitis B surface antibody; HBeAg, hepatitis B e antigen; HBeAb, hepatitis B e antibody; HBcAb, hepatitis B core antibody; PLT, platelets; WBC, white blood cell; NEUT, neutrophils; LYMPH, lymphocytes; RBC, red blood cell; HGB, hemoglobin; HCT, Hematocrit; MCV, mean red blood cell volume; MPV, mean platelet volume; HBV DNA was calculated by denary logarithm expressed as lg (HBV DNA)*.

### Fibrosis Stage F, NAS Scores, and FibroScan Results

**Figure 5:**
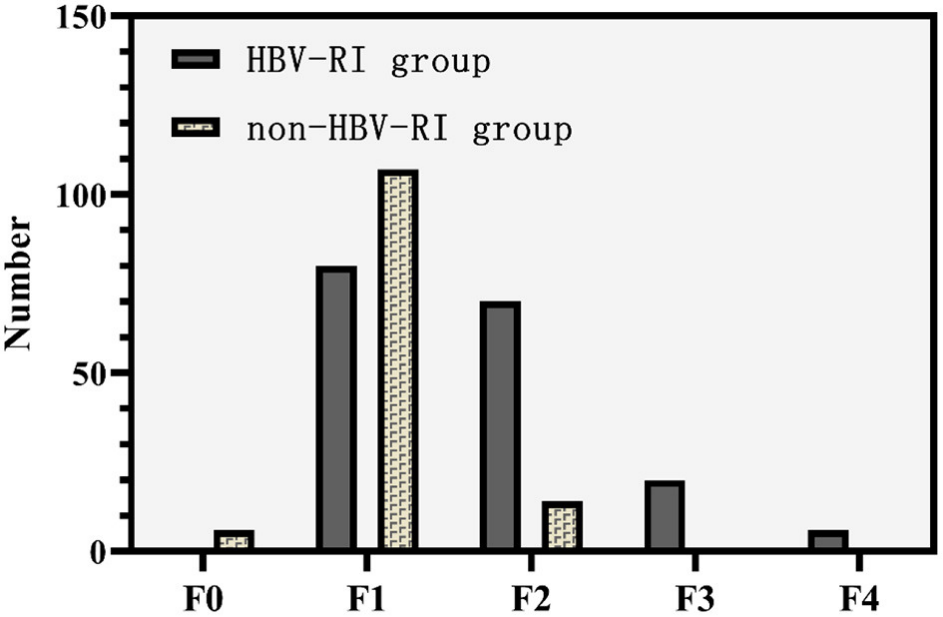
Fibrosis stage F between the HBV-RI group and the non-HBV-RI group. HBV-RI group, HBV-related inflammation group; non-HBV-RI group, non-HBV-related inflammation group; HBV-related inflammation group: F0: 0 cases, F1: 80 cases, F2: 70 cases, F3: 20 cases, F4: 4 cases; non-HBV-related inflammation group: F0: 6 cases, F1: 107 cases, F2: 14 cases, F3: 0 cases, F4: 0 cases.

**Figure 6:**
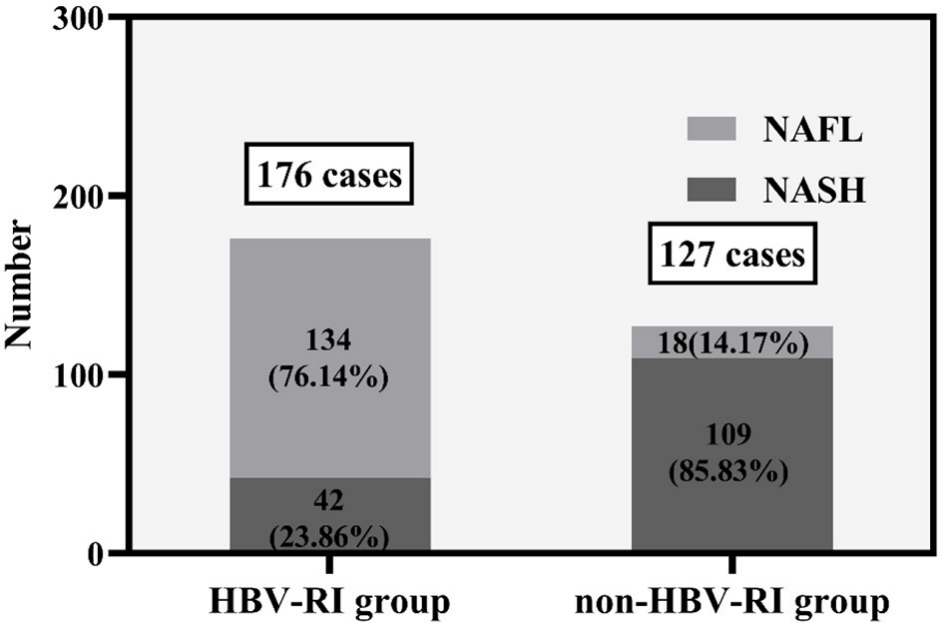
The NAS scores between the HBV-RI group and the non-HBV-RI group. HBV-RI group, HBV-related inflammation group; non-HBV-RI group, non-HBV-related inflammation group; NAFL, nonalcoholic fatty liver; NASH, nonalcoholic steatohepatitis (NASH).

**Figure 7:**
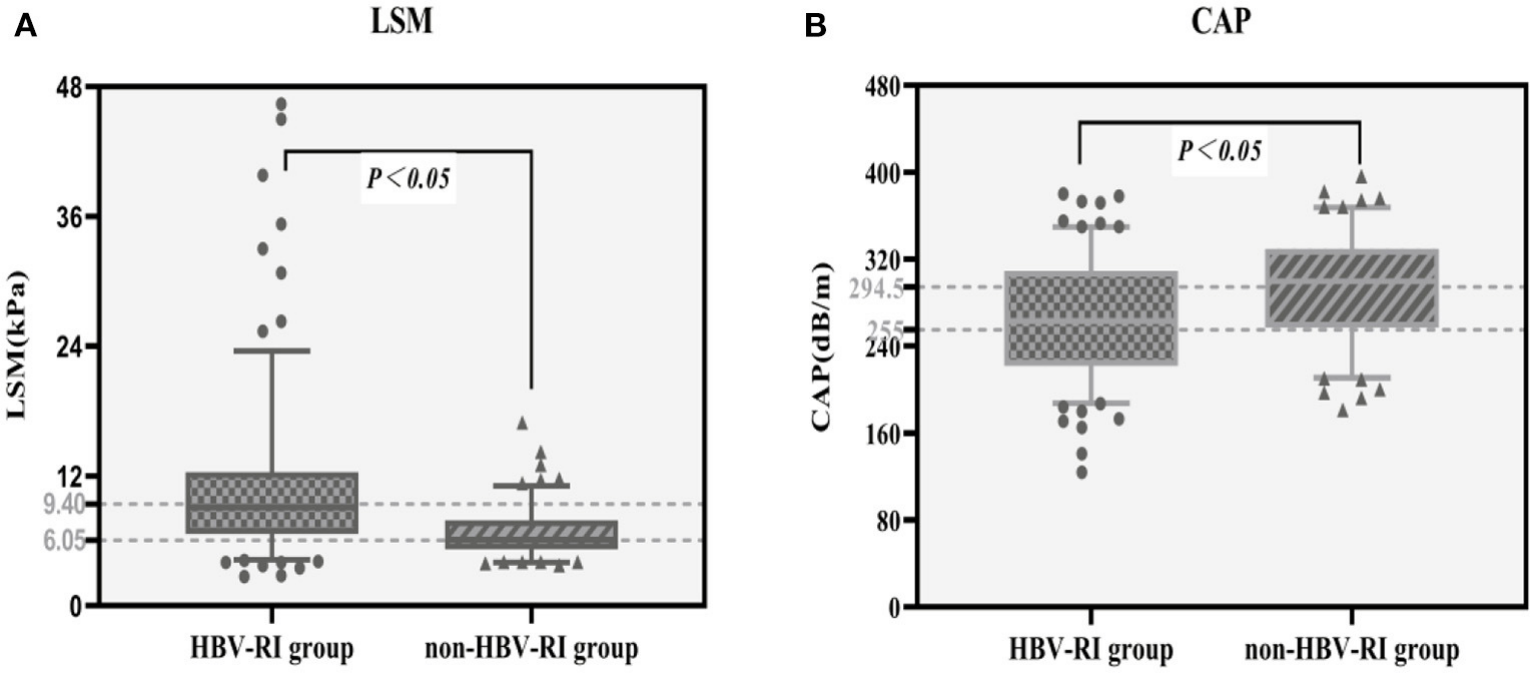
LSM and CAP results between the HBV-RI group and the non-HBV-RI group. **(A)** LSM between the HBV-RI group and the non-HBV-RI group. **(B)** CAP between the HBV-RI group and the non-HBV-RI group. HBV-RI group, HBV-related inflammation group; non-HBV-RI group, non-HBV-related inflammation group; LSM, Liver stiffness measurement; CAP, controlled attenuation parameter. The median value of LSM in the HBV-related inflammation group was 9.40 KPa, whereas in the non-HBV-related inflammation group was 6.05 U/L, and the difference was significant (*P* < 0.05); The median value of CAP in the HBV-related inflammation group was 255 dB/m, while in the non-HBV-related inflammation group was 294.5 dB/m, which was statistically significant (*P* < 0.05).

The fibrosis stage F of the HBV-I group was significantly higher than the non-HBV-I group (χ^2^ = 67.061, *P* < 0.001) (Figure 5). In the HBV-I group (176 cases), a total of 42 patients were concurrent with NASH (NAS of >4), while in the non-HBV-I group (127 cases), as many as 109 cases (χ^2^ = 113.294, *P* < 0.001) (outlined in Figure 6). LSM in the non-HBV-I group (6.74 ± 2.25) kPa was significantly lower than the HBV-I group (10.70 ± 6.90) kPa (*t* = 6.974, *P* = 0.000), while the CAP value was markedly higher in the non-HBV-I group than the HBV-I group (295.27 ± 46.73 vs. 265.37 ± 51.84) dB/m (*t* = −5.039, *P* = 0.000) (Figure 7).

### Development of a Novel Noninvasive Diagnostic Model of HBV-Related Inflammation

**Figure 8:**
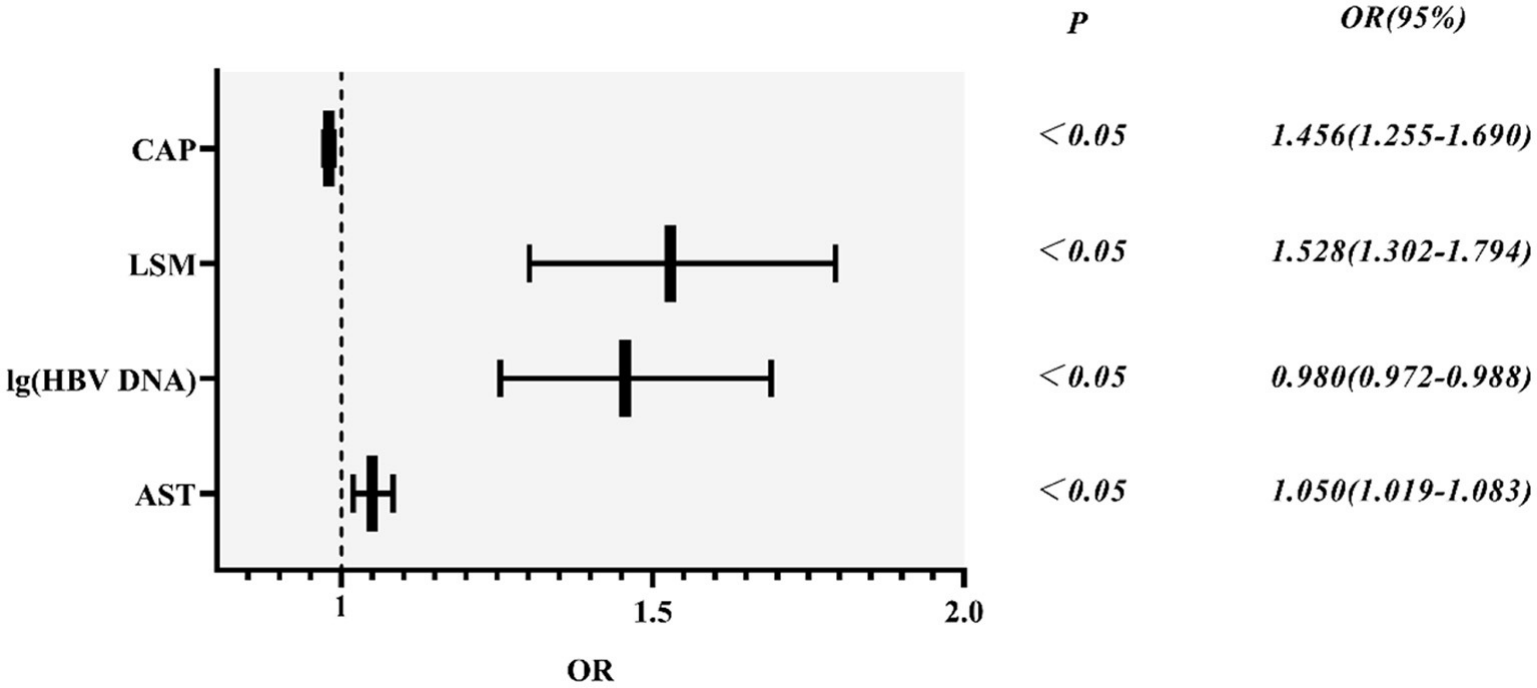
HBV-related inflammation forest diagram in CBI patients with concurrent NAFLD. CBI, chronic HBV infection; NAFLD, non-alcoholic fatty liver disease; CAP, controlled attenuation parameter; LSM, Liver stiffness measurement; HBV DNA was calculated by denary logarithm expressed as lg (HBV DNA); AST, aspartate aminotransferase; OR/Exp(B): odds ratio.

**Figure 9:**
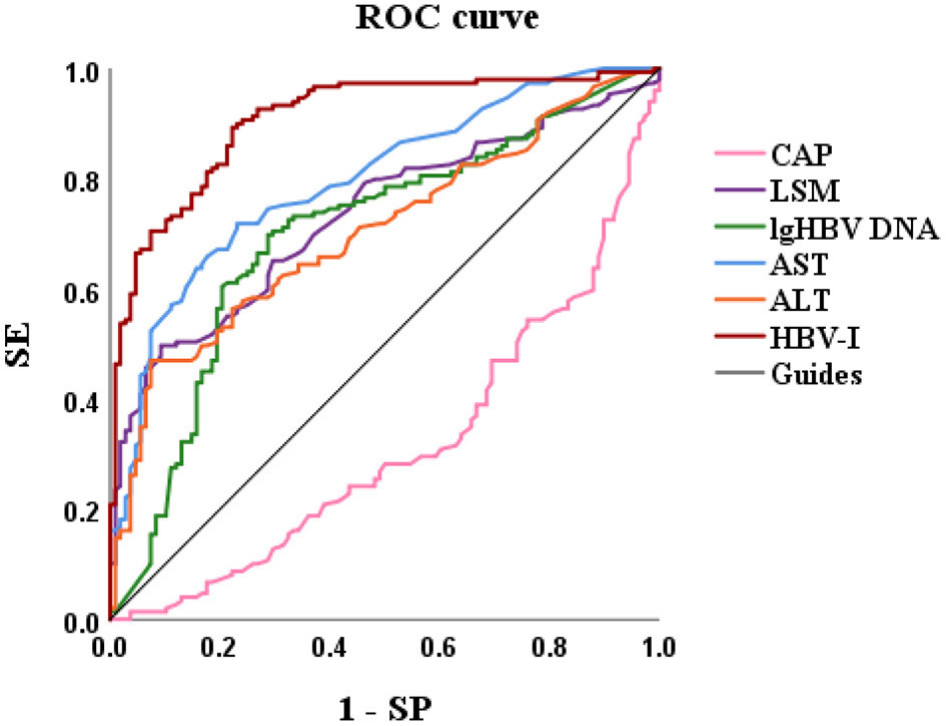
The AUROCs of HBV-I, CAP, LSM, lg (HBV DNA), AST, and ALT for HBV-related inflammation of CBI patients with concurrent NAFLD. AUROC, area under the receiver operating characteristic curve; HBV-I, hepatitis B virus inflammation model; LSM, Liver stiffness measurement; HBV DNA was calculated by denary logarithm expressed as lg (HBV DNA); CAP, controlled attenuation parameter; LSM, Liver stiffness measurement; AST, aspartate aminotransferase; ALT, alanine aminotransferase; CBI, chronic HBV infection; NAFLD, nonalcoholic fatty liver disease; SE, sensitivity; SP, specificity; the AUROCs in HBV-I, CAP, LSM, lg (HBV DNA), AST, ALT were 0.907, 0.314, 0.732, 0.699, 0.797 and 0.704, respectively.

Based on variables with a *P*-value < 0.2 on the univariate analysis, the multivariate logistic regression analysis showed that only CAP, LSM, AST, and lg (HBV DNA) were independent influencing factors, and the difference was statistically significant (*P* < 0.05). A novel equation established through logistic regression: HBV-I = −0.020 × CAP + 0.424 × LSM + 0.376 × logDNA + 0.049 × AST, with an accuracy of 82.5%. Further analysis of the diagnostic efficacy of HBV-I showed that the AUROC is 0.907, the cutoff value is 0.671, the sensitivity (SE) is 89.30%, the specificity (SP) is 77.80%, the positive predictive value is 90.34%, and the negative predictive value is 81.89% (Table 3; Figures 8, 9). The AUC values of each risk factors, as well as ALT, are shown in Figure 9.

**Table 3:** The logistic regression analysis of HBV-related inflammation in CBI patients with concurrent NAFLD.

**Variables**	**B**	**Standard error**	**Wals**	***P*值**	**Exp(B)**	**95%CI**
CAP	−0.020	0.004	23.52	0.000	0.980	0.972-0.988
LSM	0.424	0.082	26.82	0.000	1.528	1.302-1.794
lg(HBV DNA)	0.376	0.076	24.45	0.000	1.456	1.255-1.690
AST	0.049	0.015	10.28	0.001	1.050	1.019-1.083
Constant	−0.458	1.500	0.093	0.760	0.633	

*HBV-RI group, HBV-related inflammation group; Non-HBV-RI group, Non-HBV-related inflammation group; LSM, Liver stiffness measurement; CAP, controlled attenuation parameter; AST, aspartate aminotransferase; HBV DNA was calculated by denary logarithm expressed as lg (HBV DNA); CI, confidence interval*.

### Validation of the Noninvasive Models of HBV-I

**Figure 10:**
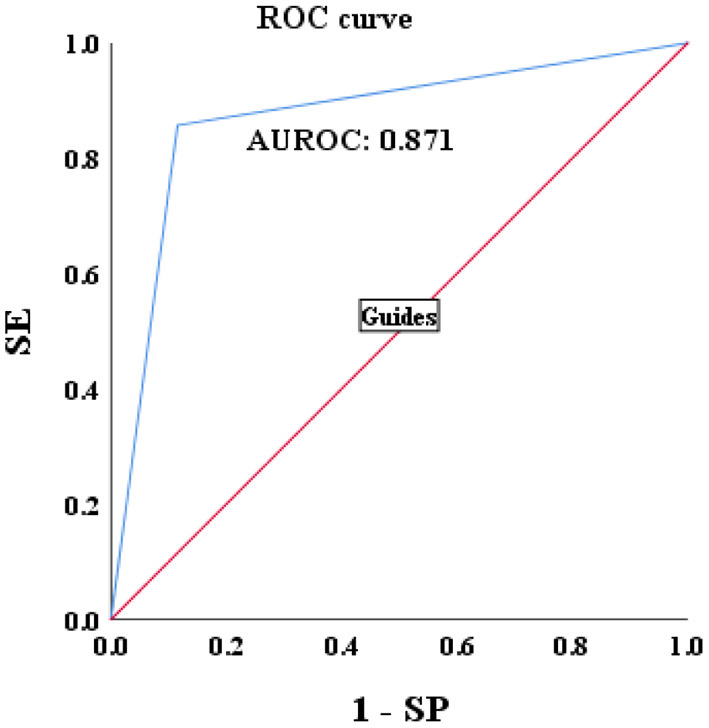
Validations of the performance for the diagnosis of HBV-I in CBI patients with concurrent NAFLD by using the AUROC. HBV-I, hepatitis B virus inflammation model; CBI, chronic HBV infection; ROC, receiver operating characteristic curve; AUROC, area under the ROC; NAFLD, nonalcoholic fatty liver disease; SE, sensitivity; SP, specificity; the AUROC in validation group was 0.871.

A total of 115 patients with CBI with concurrent NAFLD who underwent liver biopsy from January 2021 to January 2022 were enrolled. Sixty three patients were biopsy-proven to HBV-I, and the others were confirmed to non-HBV-I by biopsy. Clinical data were collected, and the HBV-I value was calculated for each patient. The formula HBV-I diagnosed 60 cases of the HBV inflammation group and 55 cases of the non-HBV-I group. Among them, nine cases of them that should be divided into the HBV-I group were classified into the non-HBV inflammation group, and the accuracy was 86.96%. Similarly, six cases were pathologically diagnosed with non-HBV-I but classified into the HBV-I group by HBV-I, and the accuracy was 85.71% (outlined in Table 4). The AUROC was 0.871, and the overall accuracy was 88.46%, as shown in Figure 10.

**Table 4:** Validation results of noninvasive diagnosis model for CBI patients with concurrent NAFLD.

**Formula**	**Overall accuracy (%)**	**Sensitivity (%)**	**Specificity (%)**	**Positive predictive value (%)**	**Negative predictive value (%)**	**AUC (95%CI)**
HBV-I	87.14%	85.30%	81.10%	83.33%	91.18%	0.868 (0.780-0.956)

*CBI, chronic HBV infection; NAFLD, nonalcoholic fatty liver disease; HBV-I, hepatitis B virus inflammation model*.

## Discussion

It has been estimated that the prevalence of biopsy-confirmed NAFLD in patients with CHB ranges from 14 to 30% (17). Lee et al. (18) studied 321 patients with CHB and showed that overlapping NAFLD could increase the risk of HCC by three times. Cardiovascular diseases (CVD) are the main cause of death of NAFLD (19), and studies (20) have confirmed that NAFLD can increase the probability of CVD in patients with CBI. NAFLD can also increase the all-cause mortality of patients with CBI patients for increasing occurrence of diabetes, hyperlipidemia, colorectal tumors, chronic kidney disease, and other diseases (5, 21). NASH is progressed from NAFL, which refers to fatty degeneration and inflammation of liver cells. Approximately, only 4% of patients with NAFL can develop cirrhosis, but more than 20% of patients with NASH can develop cirrhosis (22). Overlapping NASH can bring a second hit for the progression of liver inflammation and liver fibrosis. Lipid overflow in hepatocytes, as major metabolic and lipid handling cells, under conditions of mitochondrial overstraining, could lead to mitochondrial and peroxisomal dysfunction and enhanced oxidative stress. Continuous chronic inflammation can cause increased extracellular matrix deposition as well as promote liver fibrosis progression due to myofibroblasts (MF) which are derived from activated hepatic stellate cells (HSC) (23, 24). CHB mainly occurs in the immune clearance and reactivation phases of patients with CBI (13). Many of these patients without significant symptoms just show mild abnormality of ALT and AST, but indeed the histology has had obvious liver inflammation which might aggravate liver fibrosis or even induced cirrhosis in case not prescribing antiviral treatment in time. The etiology of inflammation in CBI concomitant with NAFLD may be CHB, NASH, or both, and the treatment depends. But the most important task is to make sure who needs anti-HBV therapy, such as nucleoside (acid) drugs and interferon-α. Inappropriate antiviral treatment with HBV concurrent with NASH brings the risk of HBV drug resistance as well as primary nonresponse and increases the economic burden of treatment, which may also influence the treatment adherence of patients with CHB (8, 25). But for NASH, in most cases, weight loss through dietary modification and exercise is the cornerstone of management. Similarly, lack of antiviral treatment in patients with CHB because the physicians mistakenly believe that inflammation is primarily from NASH carries the risk of progressive liver fibrosis, cirrhosis, and HCC (26, 27). This study (28) suggested that NAFLD might affect the biochemical response rather than the virological response. Although the effect of fatty liver on the efficacy of anti-HBV therapy is controversial (29), no research believes that reducing fatty liver has adverse effects on anti-HBV therapy, and active treatment of fatty liver will not only reduce fatty liver-related diseases, such as diabetes, hyperlipidemia, and atherosclerosis, but also reduce the incidence of HCC and all-cause mortality. Therefore, we are supposed to pay attention to dealing with fatty liver in CBI patient concomitant with NAFLD in any condition. Both CHB and NASH contribute to increased ALT levels, thus, the ALT level cannot distinguish liver inflammation appropriately. In addition, in this study, although the ALT level of the HBV-I group was significantly higher than that of the non-HBV-I group, it is not an independent factor for HBV-I in CBI patients with concurrent NAFLD in logistic regression analysis. Currently, the American Association for the Study of Liver Diseases (AASLD) (30), European Association for the Study of the Liver (EASL) (31), and other guidelines on CHB have not clearly proposed the diagnosis and treatment of CBI concurrent with NAFLD. At present, CHB and NAFLD are mainly distinguished by liver biopsy, however, there are no simple and clinically easy-to-operate experimental indicators for reference. In addition, liver histopathology is invasive, expensive, difficult-to-repeated and has sampling error, what is more, it is difficult to be implemented in primary hospitals (32). Thus, it is urgent to establish an easy-to-clinical, effective, economical and noninvasive diagnostic model to predict accurately the HBV-I of CBI concurrent with NAFLD so as to use anti-HBV treatment. Liang et al. (25) showed that cytokeratin 18(CK-18) fragment M30 has reference significance for identifying the NASH-related inflammation of CHB concurrent with NAFLD, and proposed a regression equation, including CK-18 M30, CAP, FBG, and HBV DNA levels, to predict NASH accurately. Xu et al. (33) developed a noninvasive diagnosis model, including reactive oxygen species (ROS), CAP, and WBC, to diagnose NASH in patients with CBI plus NAFLD. Liu et al. (34) found that the diagnostic value of miRNA-34a for NASH (AUROC = 0.811) is superior to that of ALT, CK-18, CK 19-M30/M65, FIB-4, and APRI in NAFLD. Nevertheless, ROS, CK, and miRNA are not clinical routine tests, so it is difficult to promote and apply in clinical practice. Furthermore, there is no simple and noninvasive diagnostic method for HBV-I in patients with CBI with concurrent NAFLD. In this article, patients with CBI with concurrent NAFLD in the inflammation are divided into HBV-I group (HAI ≥ 4) and non-HBV-I group (HAI < 4) according to liver histopathology, and a noninvasive diagnosis model of HBV-I was established and verified based on demographic, serological, and imaging indicators.

In this study, the proportion of men in the non-HBV-I group was higher than that in the HBV inflammation group. As we know, the primary causes of NASH (35) involve living a sedentary lifestyle, eating an overdose of calories, etc. which may be because men apt to have bad living habits, such as high-fat diets and staying up late than women. In addition, Ballestri et al. (36) believed that estrogen is deemed to be a protective factor to prevent fatty liver, and thought that estrogen supplementation may at least theoretically protect from NAFLD development and progression. These may be the reasons for the higher proportion of men in the non-HBV-I group. ALT, AST, γ-GT, ALP in the HBV inflammation group were significantly higher than those in the non-HBV-I group (*P* < 0.05), while ALB was significantly lower than in the non-HBV-I group (*P* < 0.05), indicating that HBV has a greater impact on liver damage in patients with CBI with concurrent NAFLD than NASH. UA and TG are important metabolic indicators in the organism. A large number of studies (36, 37) showed that abnormal UA is a high-risk factor for a variety of metabolic diseases. Kunutsor's et al. (37) research suggests that hyperuricemia is closely associated with NAFLD, metabolic syndrome, chronic cardiovascular events, and chronic kidney disease, which is an independent risk factor for metabolic syndrome and chronic cardiovascular events; Sertoglu et al. (38) confirmed that hyperuricemia is an independent risk factor for hepatocyte ballooning in patients with NASH. Yang et al. (11) found that TG has a good diagnostic value for NASH in patients with CHB and NAFLD, indicating the accumulation of liver lipids. In general, insulin resistance plays a key role in liver lipid imbalance, and the accumulation of TG in the liver will aggravate liver insulin resistance, thereby increasing insulin-antagonistic cytokines (39). Chiang et al. proved (40) that patients with CBI have lower serum TG and higher serum adiponectin (ADPN) levels. The UA and TG of the non-HBV-I group were significantly higher than those of the HBV inflammation group, which is consistent with previous studies, indicating that metabolic factors have a greater impact on the non-HBV-I group. It is suggested that early interventions, such as a low-fat diet and exercise, can be carried out to improve the prognosis. HBV DNA is mainly used to assess the level of virus replication in HBV-infected individuals, and it contributes to the selection of indications and efficacy of antiviral therapy. In the process of antiviral therapy, a sustained virological response can significantly suppress virus replication and reduce disease progression (41, 42). HBV DNA positive means that a large amount of hepatitis B virus with active replication and strong infection occurs in an organism, but it is uncorrelated directly with liver disease and only results from qualitative examination of HBV. Additionally, antiviral treatment should be determined according to the level of ALT, liver inflammation, and fibrosis degree in the body (13). Studies by Mi et al. (43), Chenet al. (44), and others found that HBV DNA of CHB patients with concurrent NAFLD was significantly lower than that of patients with CHB alone, indicating that NAFLD may inhibit viral replication. In this article, HBV DNA was calculated by denary logarithm, and lg (HBV DNA) in the HBV inflammation group was significantly higher than that in the non-HBV-I group (*P* < 0.05), which may happen because more active virus replication occurs in the HBV-I group. But there are also some individuals in the HBV-I group with persistent low DNA and abnormal transaminase, and it is known that NAFLD itself can also have abnormal transaminase (11). Therefore, it is necessary to pay attention to the level of HBV DNA in patients with HBV infection when the transaminase is elevated, select patients in the immune clearance period suitable for treatment, and identify other potential liver damage factors which are irrelevant to HBV-infected. In this study, HBeAg in the HBV-I group was higher than that in the non-HBV-I group, indicating that most patients in the HBV-I group were in the immune clearance period, which takes on HBeAg positive, while the non-HBV-I group could mostly be in the immune tolerance period or low activity period.

Thyroid hormones (THs) play an important role in fat mobilization, lipolysis, and lipid oxidation. A variation in thyroid function parameters may be associated with atherosclerosis and cardiometabolic diseases (45). TH plays a decisive role in regulating energy homeostasis and insulin resistance (46). NAFLD and NASH are closely related to energy homeostasis disorder and insulin resistance (47). Therefore, TH could be involved in the pathogenesis of NAFLD and NASH. In recent years, many studies (48, 49) have shown that thyroid dysfunction is related to NAFLD. It is estimated (47–50) that worldwide, the incidence of hypothyroidism among patients with NAFLD ranges from 15.2 to 36.3%. Xu et al. (51) reported that TSH in patients with NAFLD was higher than that in patients without NAFLD, whereas T4 level was lower. Liu et al. (52) divided CHB patients with concurrent NAFLD into the NASH group and the non-NASH group and concluded that TSH was higher in the NASH group, but FT3 and FT4 were lower in the NASH group. The animal experiments of Chi et al. (53) confirmed that a large amount of TT3 in mice could inhibit the expression of hepatitis B protein x (HBx) to eliminate the production of ROS, hence, reducing liver cell death by HBx and liver inflammation in mice. Chi et al. (54) pretreated different exposure concentrations of TH in another animal experiment and proved that the protective effect of TH on HBx-induced liver cancer is achieved by reducing the induction of HBV-DNA on ROS. In this article, the levels of TT3 and TT4 in the non-HBV-I group were lower than those in the HBV-I group (*P* < 0.05), which was consistent with the above research results, while there was no significant difference in TSH between the two groups which may be affected by viral factors. The levels of TT3 and TT4 in the HBV-I group are slightly higher than normal level to maintain a relatively high metabolic state, and then affect the glucose as well as lipid metabolism state of the body, which could also be the potential mechanism of HBV infection to reduce the probability of lipid metabolism disorders in patients with NAFLD. It has been found (51) that certain exogenous thyroid hormones and thyroid hormone analogs contribute to improving the degree of liver steatosis in patients with NAFLD. Finan et al. (55) found that the hybrid thyroid hormone-glucagon 147 complex can improve the degree of liver steatosis in NAFLD patients without adverse reactions in the skeletal muscle and cardiovascular system. The synthesis of this novel compound has opened up a new field for the simultaneous dual-hormone treatment of multiple metabolic diseases. The above studies indicate that TH may be the key to the treatment of certain metabolic diseases, and in the future, the substitutes or mimics of TH may be applicable to treat metabolic diseases, such as hyperlipidemia and NAFLD.

CHB is a liver immune pathological damage based on mediated by T lymphocytes. When CHB occurs, liver cells are affected by viral stimulation and activate the immune response. Cytokines can induce specific T lymphocytes to kill liver cells, causing diffuse degeneration and necrosis of liver cells, destruction of the original liver lobule structure, and collapse of the fiber scaffold, all of which can lead to fibrous tissue proliferation and nodular regeneration of hepatocytes, thereby these cells could lose secretion function, resulting in a decrease in serum complement levels (56). A study has found (57) that liver injury in patients leads to decreased immune globulin clearance and dysfunction of Kupffer cells in hepatic sinusoids to prevent non-specific phagocytosis and removal of self or foreign antigens, thus increasing the level of immune globulin. Domestic scholar Guo (58) found that the severity of CHB is positively associated with levels of IgA, IgG, and IgM. Another study has shown (59) that C3 is an important predictor of metabolic syndrome, insulin resistance, and hypertension. A Dutch cross-sectional study (60) selected 523 middle-aged and elderly people with increased risk of metabolic and CVD, and found that the increase of C3a was positively correlated with liver fat content. In this article, C3 in the HBV inflammation group was lower than that in the non-HBV-I group (*P* < 0.05), while IgG and IgA were higher than the non-HBV-I group (*P* < 0.05), which was consistent with the above research results. Beibei et al. (61) believed that patients with CHB concurrent with NAFLD have weakened immunity to viral attacks, which may be one of the reasons for the poor effect of interferon therapy.

The RBC parameters of patients with CHB were found (62) in relation to liver function. On the one hand, massive necrosis of liver cells brings a sharp decline in intake and storage of vitamin B12, folic acid, and iron, which impedes the growth and development of RBCs and even leads to megaloblastic anemia. On the other hand, during the active phase of CHB, immune system disorders aggravated liver cell necrosis, increasing tumor necrosis factor, endotoxin, and various cytokines (such as interleukin-1 and interleukin-6). That not only contributes to the development of the bone marrow hematopoietic microenvironment and hematopoietic stem cells (63) but also directly acts on the mature RBCs in the peripheral blood to change the RBC membrane structure, which both transforms the antigenicity of the RBCs surface and sensitize the RBCs, therefore, easily recognized phagocytic and destroyed by macrophages of the spleen macrophage system (64, 65). This is consistent with the results in this study, i.e., the RBC of the HBV inflammation group is lower than the non-HBV-I group, and the MCV is higher than the non-HBV-I group (*P* < 0.05). Clinically, MPV is often used to judge bleeding tendency and the status of bone marrow hematopoietic function. Karagoz et al. (66) showed that the MPV in patients with CHB is elevated, and the elevated level of MPV is related to the severity of CHB and liver fibrosis. The mechanism of HBV infection inducing the increase of MPV levels might be as follows: when hepatocytes are infected with HBV, they can produce and release a large number of inflammatory mediators, which can induce a large number of immature PLTs to enter the hematopoietic circulation pool of bone marrow, thus causing the increase of MPV level in parents (67). Arslan and Makay (68) pointed out that MPV could be used as a predictor of atherosclerosis in obese patients with NAFLD, and it is often accompanied by an increase in MPV in patients with NAFLD. Saremi et al. (69) found that compared with healthy controls, the level of MPV in patients with NAFLD was significantly higher. Alkhouri et al. (70) confirmed that with biopsy-proven NAFLD, MPV values were higher for NASH than for NAFL and normal biopsy. In this study, the MPV of the HBV-I group was higher than that of the non-HBV-I group (*P* < 0.05), which may be due to the greater impact of viral factors on MPV and the higher degree of liver fibrosis in the HBV-I group.

FibroScan (FS) can achieve accurate diagnosis and evaluation of liver fibrosis and cirrhosis, dynamic monitoring of anti-fibrosis efficacy, and prediction of complications of liver cirrhosis by measuring LSM. Currently, it has been extensively used in advanced liver fibrosis and cirrhosis caused by CHB (19, 26) and NAFLD (43). CAP is a new parameter set based on the principle of ultrasound attenuation developed by FS. It is preliminarily confirmed that CAP can effectively detect ≥ 10% steatosis, higher than that of B-ultrasound or CT, which can be used for quantitative evaluation of hepatic steatosis (71). In this study, the LSM value of the HBV-I group was significantly higher than the non-HBV-related inflammation group (*P* < 0.05), which is consistent with the result that liver fibrosis was significantly higher than the non-HBV-related inflammation group, while CAP of the HBV inflammation group was lower than the non-HBV-I group (*P* < 0.05). Chen et al. (44) found that the LSM value of patients with CHB concurrent with NAFLD was significantly higher than that of patients with CHB alone (*P* < 0.05). Liang et al. (25) found that the CAP value of patients with NASH plus CBI was significantly higher than that of patients with NAFL plus CBI (*P* < 0.05). Mi et al. (43) confirmed that CAP has a good diagnostic value for hepatic steatosis in CBI patients with concurrent NAFLD, especially for patients with severe hepatic steatosis. The results of the meta-analysis of Karlas et al. (72) also showed that CAP provided a standardized noninvasive measurement method for the diagnosis of liver steatosis in patients with CHB, but the result also shows that the contributing factors, such as the etiology of liver disease, diabetes, and BMI, should be considered when interpreting the CAP value. Xu et al. (73) suggested that CAP has a higher detection rate for hepatic steatosis of the CBI patients with concurrent NAFLD than ultrasound, which is less sensitive to hepatic steatosis and could only detect ≥ 30% of hepatic steatosis. These patients mostly have mild fatty liver, so ultrasound has a high rate of misdiagnosis of NAFLD in patients with CHB. Moreover, CAP is an ideal tool for the diagnosis of fatty liver, but there is also the problem of overestimating the degree of fatty liver.

In this study, significant factors in the univariate analysis (*P* < 0.2) were subjected to stepwise regression analysis and we could obtain a formula: HBV-I = −0.020 × CAP + 0.424 × LSM + 0.376 × lg (HBV DNA) + 0.049 × AST, with 82.5% accuracy. The AUROC was 0.907, the cutoff value was 0.671, the sensitivity was 89.30%, the specificity was 77.80%, the positive predictive value was 90.34%, and the negative predictive value was 81.89%. It is suggested that for patients with CBI with concurrent NAFLD in inflammation, the higher LSM value, the higher lg (HBV DNA), the higher AST, the greater the possibility of active HBV infection, while the higher the CAP value implied the heavier the proportion of NASH. In previous studies, AST was mainly considered as a part of noninvasive diagnostic models, such as APRI and FIB-4, to diagnose liver fibrosis (74), and was rarely used in the diagnosis of CHB or NASH. In this study, AST is an independent influencing factor for CHB concurrent with patients with NAFLD and the AUROC of AST is 0.797, which has certain diagnostic performance for HBV-I. The AUROC of LSM, CAP, and lg (HBV DNA) were 0.732, 0.314, and 0.699, respectively. The results of the ROC curve suggest that whether it is the liver function index (AST) or the virological index (HBV DNA) that we pay more attention to, or the FibroScan, which has been more clinically studied in recent years, it has certain significance for disease prediction, but there are limitations for single indicator, and possibly several factors affect the results. In total, 115 patients were enrolled into the validation group and their clinical data were verified by HBV-I, and the results show that the accuracy was 86.96% and the AUROC was 0.871, indicating that HBV-I has a good clinical value and repeatability. There are validation errors in the validation group (15 cases): 9 cases which proven by a biopsy to belong to HBV-I were deemed to non-HBV inflammation group by mistake, and we call it the false-negative group; 6 cases of pathological non-HBV-I is verified as the HBV-I group incorrectly, which is called the false positive group. There are 5 individuals in the false-negative group with a CAP value > 290 dB/m. Considering the influencing factors of CAP, many studies (72, 75, 76) have proved that CAP is related to subcutaneous fat thickness, BMI, age, and other factors. Xu et al. (73) proposed that CAP may overestimate the level of liver steatosis and Satoshi et al. (75) believed that it can only improve the accuracy of the LSM value instead of CAP by calculating IQR and taking the average of 10 FS detection values. The AUROC of CAP is 0.314, indicating a poor predicting effect alone, which is also consistent with the result of high CAP in five cases of false negative in the validation group. Studies found (75, 77) that FS probes can also have different CAP values and LSM values. FS probes are mainly divided into two types, an M probe is used for most patients, and an XL probe is designed for patients with obesity. The ultrasonic center frequency of the M probe is 3.5 MHz, and the XL probe is 2.5 MHz. Furthermore, it is recommended to use the M probe for any patients with a skin-envelope distance (SCD) < 25 mm, and the XL probe for patients with an SCD > 25 mm. In the same population, the LSM value obtained with the M probe is higher than the XL probe. Previous studies have shown (77, 78) that the CAP value returned by the XL probe is higher than that of the M probe. In this study, an M probe used in all patients was used to detect LSM and CAP. However, among the patients, there were some patients with BMI > 30 kg/m^2^, resulting in higher LSM value measurement and lower CAP than actual values possibly, which may affect the results of the experiment. Some scholars improve the diagnostic efficiency of fatty liver by improving CAP technology. Fujiwara et al. (79) used the C1-6-D phased array probe to detect the LOGIQ E9 XD clear 2.0 ultrasound (GE Healthcare, USA) guided attenuation parameter (UGAP) detection and the AUROC of the UGAP-values for identifying S1, S2, and S3 of hepatic steatosis (0.900, 0.950, and 0.959) is significantly better than the AUROC of CAP values (0.829, 0.841, and 0.817), respectively. Ren et al. (80) measure liver steatosis analysis (Lisa) with Hepatus, a newly developed ultrasound machine, with a 3.5-MHz phased array probe (LFP5-1U, Mindray, Re 6S, China) on 203 patients with CHB. Compared with CAP detected by FS probe, the diagnostic efficiency of Lisa for steatosis ≥ 10% was significantly better than that of CAP (AUC: 0.859 vs. 0.801, *P* < 0.05). Among the six false-positive patients, the AST values in three patients were significantly higher than the average value of AST in biopsy-proven non-HBV-related patients and were higher than twice the ULN for AST, indicating that liver dysfunction occurs on those, and the damage is possibly caused by NASH rather than CHB. In addition, CAP values in the other two patients (202 and 217 dB/m) were lower than the average value of the noninflammatory group (265.37 dB/m) causing a formula error probably. For rest of the patients, the LSM value was 9.3 kPa, and the CAP was 357 dB/m. Salvatore et al. (19) believed that CAP can weaken the diagnostic efficiency of the LSM value, leading to LSM may overestimate the severity of liver fibrosis. Studies (81–83) believe that the influencing factors of LSM value include inflammation, venous pressure, cholestasis, and amyloid deposition. The administration of diuretics to patients with high venous pressure can also reduce the LSM value (82). We can further expand the sample size of the validation group, or improve the FS, to strengthen the diagnostic efficacy of the HBV-I formula so that it can obtain greater clinical application value.

This prospective clinical study is not exempt of limitations: first, this study included too few cases of severe inflammation (HAI ≥ 10) and liver fibrosis (F ≥ 3) in the modeling group and validation group; second, the liver function of the patients in this study was mild to moderate abnormality, demonstrating that is the application of HBV-I, and whether it is applicable to patients outside this range needs to be discussed; third, results may not apply to populations characterized by a different risk profile or to children, other ethnicities, etc., as these types of patients are not included in this study; finally, this study was performed based on data from a single-center study, and a multi-center study (84) is supposed to be conducted to reduce the study bias.

## Data Availability Statement

The original contributions presented in the study are included in the article/supplementary material, further inquiries can be directed to the corresponding author/s.

## Ethics Statement

The studies involving human participants were reviewed and approved by the Medical Ethics Committee of Tianjin Second People's Hospital. The patients/participants provided their written informed consent to participate in this study.

## Author Contributions

XT, LX, and YM designed the study. XT, LC, YZ, BJ, and LX performed the research. YL and RS made the pathological diagnosis. XT carried out statistical analysis. XT, LC, and LX wrote the manuscript. YM critically revised the manuscript. All authors have read and approved the final revision to be published.

## Funding

This work was supported by Chinese Foundation for Hepatitis Prevention and Control—Tian Qing Hepatitis Research Fund, No. TQGB20210175, Research project of Chinese traditional medicine and Chinese traditional medicine combined with Western medicine of Tianjin municipal health and Family Planning Commission (2015061/2017070/2021022), and Tianjin Key Medical Discipline (Specialty) Construction Project.

## Conflict of Interest

The authors declare that the research was conducted in the absence of any commercial or financial relationships that could be construed as a potential conflict of interest.

## Publisher's Note

All claims expressed in this article are solely those of the authors and do not necessarily represent those of their affiliated organizations, or those of the publisher, the editors and the reviewers. Any product that may be evaluated in this article, or claim that may be made by its manufacturer, is not guaranteed or endorsed by the publisher.
